# Electroacupuncture Pretreatment Elicits Tolerance to Cerebral Ischemia/Reperfusion through Inhibition of the GluN2B/m-Calpain/p38 MAPK Proapoptotic Pathway

**DOI:** 10.1155/2020/8840675

**Published:** 2020-09-29

**Authors:** Bao-yu Zhang, Guan-ran Wang, Wen-hua Ning, Jian Liu, Sha Yang, Yan Shen, Yang Wang, Meng-xiong Zhao, Li Li

**Affiliations:** ^1^First Teaching Hospital of Tianjin University of Traditional Chinese Medicine, Tianjin, China; ^2^Heilongjiang University of Chinese Medicine, Harbin, China; ^3^National Clinical Research Center for Chinese Medicine Acupuncture and Moxibustion, Tianjin, China; ^4^Key Laboratory of Cerebropathy Acupuncture Therapy of State Administration of Traditional Chinese Medicine, Tianjin, China; ^5^Tianjin Key Laboratory of Acupuncture and Moxibustion, Tianjin, China; ^6^Tianjin Key Laboratory of Translational Research of Prescription and Syndrome, Tianjin, China; ^7^Academy for Advanced Interdisciplinary Studies, Peking University, Beijing, China

## Abstract

**Background:**

As one of the first steps in the pathology of cerebral ischemia, glutamate-induced excitotoxicity progresses too fast to be the target of postischemic intervention. However, ischemic preconditioning including electroacupuncture (EA) might elicit cerebral ischemic tolerance through ameliorating excitotoxicity.

**Objective:**

To investigate whether EA pretreatment based on TCM theory could elicit cerebral tolerance against ischemia/reperfusion (I/R) injury, and explore its potential excitotoxicity inhibition mechanism from regulating proapoptotic pathway of the NMDA subtype of glutamate receptor (GluN2B).

**Methods:**

The experimental procedure included 5 consecutive days of pretreatment stage and the subsequent modeling stage for one day. All rats were evenly randomized into three groups: sham MCAO/R, MCAO/R, and EA+MCAO/R. During pretreatment procedure, only rats in the EA+MCAO/R group received EA intervention on GV20, SP6, and PC6 once a day for 5 days. Model preparation for MCAO/R or sham MCAO/R started 2 hours after the last pretreatment. 24 hours after model preparation, the Garcia neurobehavioral scoring criteria was used for the evaluation of neurological deficits, TTC for the measurement of infarct volume, TUNEL staining for determination of neural cell apoptosis at hippocampal CA1 area, and WB and double immunofluorescence staining for expression and the cellular localization of GluN2B and m-calpain and p38 MAPK.

**Results:**

This EA pretreatment regime could improve neurofunction, decrease cerebral infarction volume, and reduce neuronal apoptosis 24 hours after cerebral I/R injury. And EA pretreatment might inhibit the excessive activation of GluN2B receptor, the GluN2B downstream proapoptotic mediator m-calpain, and the phosphorylation of its transcription factor p38 MAPK in the hippocampal neurons after cerebral I/R injury.

**Conclusion:**

The EA regime might induce tolerance against I/R injury partially through the regulation of the proapoptotic GluN2B/m-calpain/p38 MAPK pathway of glutamate.

## 1. Introduction

Stroke is the second leading cause of death and the third leading cause of disability worldwide [1]. Stroke is estimated to cause 12 million deaths per year by 2030 [2]. Ischemic stroke, induced by occlusion of cerebral blood flow mainly in the middle cerebral artery (MCA), accounts for about 87.9% of all strokes and often leads to severe central nervous system injury or even death [3]. Currently, recombinant tissue plasminogen activator (rt-PA) is the only FDA-approved agent for the hyperacute phase of ischemic stroke [2]. However, application of such therapy is limited by the narrow therapeutic time window (3-4.5 h) and strict indications [3]. Therefore, only 2-5% of patients attacked by acute ischemic stroke have received rt-PA treatment [2]. Furthermore, rt-PA-treated patients had an absolute risk of symptomatic intracerebral hemorrhage at 2.3% per year over 10 years [4, 5]. Moreover, there was no significant reduction in mortality at 3 to 6 months after intravenous thrombolysis compared with the aspirin-control group (17.9% vs. 16.5%), and two-thirds of survivors still suffer from certain disability [6].

Since current status of stroke management has always been far below expectation, stroke neurobiologists have advanced a considerable body of evidence supporting the hypothesis that, with certain preconditioning, the mammalian brain can adapt to injurious insults such as cerebral ischemia to promote cell survival in the face of subsequent injury [7]. Such neuroprotective effect triggered by certain “preconditioning” stimuli has been referred as “cerebral ischemic tolerance.”

The candidate stimuli should be a nonlethal stimulus strong enough to initiate a response inducing tolerance against subsequent lethal attacks but not so much as to cause permanent tissue damage [8]. Various preconditioning paradigms such as hypoxia, nonlethal ischemia, and pharmacological anesthetics have been explored for providing a unique window into the brain's endogenous protective mechanisms [9]. Most of the pretreatment methods are double-edged, producing nonlethal harmful stimuli to the body for ischemic tolerance. Remote ischemic pretreatment might be the only regime that has been studied in clinical research. However, this multicenter clinical trial conducted in 2015 suggested that remote ischemic pretreatment did not improve patient outcomes [10].

Even though acupuncture and moxibustion had been applied for disease prevention since ancient time, the first evidence supporting its role for inducing cerebral ischemic tolerance in an animal model was provided by Xiong's team in 2003 [11]. The study confirmed that electroacupuncture (EA) at GV20 before cerebral ischemia can reduce the degree of postischemic neural deficit in an animal model [11]. Until now, the mechanism of cerebral ischemic tolerance induced by EA pretreatment has been explored from various aspects, such as inhibition of inflammation, oxidative stress, endoplasmic reticulum stress, regulation of cannabinoid system, autophagy, protection of blood-brain barrier, and antiapoptosis [12–14]. Nevertheless, superiority of precondition should contribute to its rapid response to lethal injury. Glutamate-induced excitotoxicity was identified as one of the first steps in the pathology of cerebral ischemia [15]. Under the pathological conditions such as cerebral ischemia, glutamate would be overreleased to the synaptic cleft and then activated the NR2B-containing NMDA receptor (GluN2B), followed by calcium overload in the cell. The overloaded calcium would activate the m-calpain in the cytoplasm and then induce the endonuclease reaction of STEP61, which further inhibit the dephosphorylation of p38 MAPKs and finally lead to cell apoptosis through signal transduction [16, 17]. The occurrence and progression of such process is too fast to be the target of postischemic intervention [18]. However, ischemic preconditioning was found to ameliorate excitotoxicity by inhibiting glutamate release [19, 20].

So, this study will focus on whether EA pretreatment could decrease excitotoxicity by regulating downstream of glutamate receptor in an animal model of cerebral ischemia/reperfusion (I/R) injury.

## 2. Material and Methods

### 2.1. Animals

Specific pathogen-free male Sprague-Dawley rats (aged 6 weeks old and weighing 250 ± 20 g) were supplied by Beijing Vital River Laboratory Animal Technology Co. Ltd. (Beijing, China; license no. SCXK(Jing)2016-0006). All rats were bred and housed in the SPF animal facility at the Institute of Radiation Medicine, Chinese Academy of Medical Sciences and Peking Union Medical College (Tianjin, China; license no. SYXK (Jin) 2019-0002) under controlled conditions in a 12-hour light/dark cycle with a ambient temperature of 20-25°C and a humidity at 40-70% for at least 3 days before preconditioning. Rats were allowed free access to a standard rodent diet and clean water. All procedures were approved by the Ethics Committee of Tianjin University of Traditional Chinese Medicine (TCM-LAEC2019018).

### 2.2. Experimental Protocol

#### 2.2.1. Experiment I: Cerebral Protective Effect of EA Pretreatment for Middle Cerebral Artery Ischemia/Reperfusion Injury (MCAO/R)

**Figure 1:**
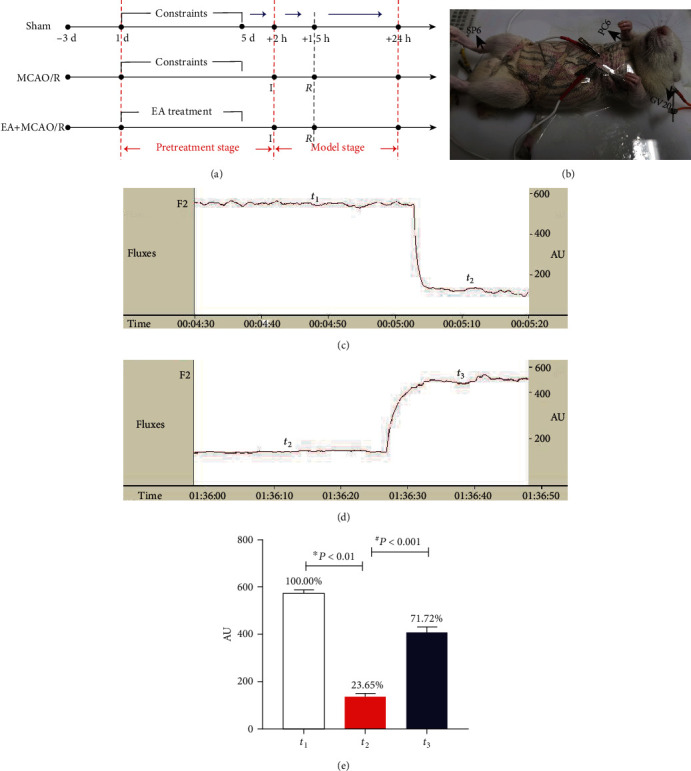
EA pretreatment and model preparation for rats in three groups. (a) The experimental procedure included 5 consecutive days of pretreatment stage and the subsequent modeling stage for one day (I: ischemia, insertion of suture to block the blood flow; R: reperfusion, withdrawal of the suture to restore blood flow at 90 minutes after ischemia). (b) EA stimulation. (c) Cerebral blood flow of the baseline (*t*_1_) and postischemia (*t*_2_). (d) Blood flow after reperfusion (*t*_3_). (e) Comparison of cerebral blood flow at different time points (*n* = 8). The unit of blood flow “AU” is the unit determined by the manufacturer. The blood flow at three time points was compared using one-way ANOVA for repeated measurement. The results did not meet the sphericity test (Mauchly *W* = 0.252, *P* < 0.05), so the differences among groups were examined using MANOVA (*F* = 470464.634, *P* < 0.001) followed by LSD for post hoc multiple comparisons. ^∗^*P* vs. sham; ^#^*P* vs. MCAO/R.

To determine the neuroprotective effect of EA pretreatment for cerebral I/R, rats were evenly randomized into three groups: sham MCAO/R, MCAO/R, and EA+MCAO/R (Figure 1(a)). The experimental procedure included 5 consecutive days of pretreatment stage and the subsequent modeling stage for one day. During pretreatment procedure, all rats were constrained by handmade cloth and only rats in the EA+MCAO/R group are receiving EA intervention. MCAO/R or sham MCAO/R procedure started after 2 hours of the last pretreatment. For all MCAO/R rats, blood flow of MCA was monitored during the modeling procedure to illustrate the validity of cerebral I/R. At 24-hour postsham-MCAO/R or MCAO/R, an independent personnel blinded to group allocation evaluates neurological deficits of all animal using the Garcia neurobehavioral scoring criteria [21]. Afterwards, all rats were sacrificed under anesthesia and the brain tissue was collected for the measurement of infarct volume or neural cell apoptosis at hippocampal CA1 area using TTC and TUNEL staining.

#### 2.2.2. Experiment II: Effect of EA Pretreatment on GluN2B and Its Downstream Pathway of MCAO/R

To determine the effect of EA pretreatment on glutamate receptor GluN2B and its downstream pathway postcerebral I/R, rats were evenly randomized into three groups: sham MCAO/R, MCAO/R, and EA+MCAO/R. All pretreatment procedures and the subsequent modeling procedure coincide with Experiment I. At 24-hour postsham-MCAO/R or MCAO/R, all rats were sacrificed under anesthesia and the right hippocampi were taken to determine the expression of GluN2B, m-calpain, and p38 MAPK by Western blot (WB) and the cellular localization of GluN2B and m-calpain using double immunofluorescence staining.

### 2.3. EA Pretreatment

During EA pretreatment, all rats were constrained by handmade cloth and fixed at the desk using adhesive tape (Figure 1(b)). Location of GV20, PC6, and SP6 in rats was referred to “Experimental acupuncture science” [22]. GV20 was selected at the midpoint between the tips of the ears and punctured backward with depth of 2 mm. Bilateral PC6 were selected 3 mm above the wrist joint on the medial side of the forelimb between the ulna and the radius and were punctured vertically with depth of 1 mm; SP6 was selected 10 mm above the tip of the medial malleolus at the hind limbs and was punctured vertically with depth of 5 mm. Five sites of acupoints were needled using sterile acupuncture needles of 0.25 mm∗25 mm (*Huatuo*, Suzhou Medical Supplies Factory, China). Then, all needles were connected to the electrodes. Unilateral PC6 and SP6 were connected to the positive and negative end of the same electrode, and GV20 and the tip of the ear were connected to the positive and negative end of the same electrode to form a current circuit using EA therapeutic apparatus (*HANS-100A*, Nanjing Jisheng Medical Technology, China). All acupoints were stimulated with the parameters of 2/15 Hz, 1 mA for 20 min/d for 5 consecutive days. Limbs and head would slightly tremor during electrical stimulation which was considered as the sign of *Deqi*.

### 2.4. The Model of Cerebral I/R Was Prepared Using Suture Method under the Monitoring of Laser Doppler Flowmeter

Focal cerebral ischemia was induced by a transient right middle cerebral artery occlusion and subsequent blood reperfusion 90 minutes later in a rat model. Before model preparation, SD rats would be fasted yet drank water freely for 12 hours. During anesthesia process, the rats were placed in the anesthesia induction chamber of the small animal anesthesia system, and the concentration of 3% isoflurane (isoflurane, RWD Life Science Co., Ltd., China) was set to induce anesthesia. After the basal reflex of the rats disappeared, the rats were fixed on the board with rubber bands with a breathing mask connected to the anesthesia system, and then, a midline incision was cut on the neck to adequately expose the right common carotid artery (CCA), external carotid artery (ECA), and internal carotid artery (ICA). After the ligation of the right CCA and ECA, a suture was inserted from the CCA into the ICA up to a depth of 18 to 20 mm. After 90 min of occlusion, the filament was withdrawn for reperfusion.

The regional cerebral blood flow (rCBF) was monitored using a Laser Doppler Flowmeter (DRT-4, Moor, UK). The cerebral blood flow after the separation of the carotid artery (*t*_1_) was set as the baseline value of blood flow. Blood flow after ischemia (*t*_2_) and reperfusion (*t*_3_) was also recorded to evaluate the effect of model preparation (Figures 1(c) and 1(d)). In each time point, when the blood flow was relatively stable, the value would be recorded and preserved for 1 min without interruption. The model was successful when local cerebral blood flow in the middle cerebral artery decreased to 30% below the baseline blood flow after ischemia [23]. The modeling process in our study could produce a representative model of cerebral I/R since local cerebral blood flow decreased to 23.65% of the baseline value after ischemia and returned to 71.72% after suture withdrawal (Figure 1(e)).

### 2.5. Garcia Score

The neurobehavioral scoring was performed according to the methods of Garcia et al. [21], which includes six aspects: spontaneous activity (in cage for 5 min, 0-3 scores), symmetry of movements (four limbs, 0-3 scores), symmetry of forelimbs (outstretching while held by tail, 0-3 scores), climbing wall of wire cage (1-3 scores), reaction to touch on either side of trunk (1-3 scores), and response to vibrissae touch (1-3 scores). Total value of Garcia is 18, with lower score for more impaired neurologic function.

### 2.6. TTC Staining

Animals were anesthetized and sacrificed to extract brain tissue. After rinsing with ice brine, the brain tissue was quickly frozen in -20°C refrigerator for 20 min to harden the brain tissue for cutting. Sections were made along the coronal plane with a thickness of 2 mm, and 5-6 slices were cut for each brain. Put the brain slices into a petri dish wrapped with tinfoil and containing 2% TTC (Solarbio, China) and then dye them in a 37°C incubator for 30 minutes. During this period, gently turn the brain slices so that they can be fully exposed to the reaction. Take out the petri dish, suck out the TTC solution with 1 ml needle tube, inject 4% paraformaldehyde solution to fix it for 5 min, and then take out and place the slices in order for pictures. Finally, the cerebral infarction volume ratio of each brain slice was calculated by Image-Pro Plus image software. In order to reduce the error caused by cerebral ischemic hemispheric edema, the infarct volume was used to subtract the ipsilateral normal tissue volume from the contralateral normal tissue volume, and the result was expressed by the percentage of infarct volume: infarct volume percentage = (contralateral tissue volume‐ipsilateral normal tissue volume)/contralateral normal tissue volume∗100%.

### 2.7. TUNEL Staining

After dewaxed by xylene and dehydrated with ethanol, paraffin sections were added with 20 *μ*g/ml protease K working solution (Solarbio, China) for 15 min at room temperature. Then, 51 *μ*l of TUNEL detection solution (45 *μ*l equilibrium buffer : 5 *μ*l nucleotide mixture : 1 *μ*l rTdT enzyme) was added to each sample and incubated in the dark for 1 h. 2× SSC was incubated at room temperature for 15 min to terminate the reaction (Everbright Inc., USA). Add DAPI containing antifluorescence quench to incubate for 5 min in the dark and then cover the coverslip. 200x images of hippocampal CA1 were obtained by a fluorescence microscope (Leica, Germany). The percentage of apoptotic positive cells in different visual fields was calculated, and the mean value was taken for statistical analysis. To represent the results of TUNEL staining, we calculated the apoptosis rate using the following formula: apoptosis rate = positive cells/total cells per field∗100%.

### 2.8. Western Blot (WB)

The total protein was lysed with histiocyte lysate. The lysate buffer was prepared as follows: 1 ml RIPA buffer containing 10 *μ*l protease inhibitor (PMSF) and 10 *μ*l protein phosphatase inhibitor (Solarbio, Beijing, China). The hippocampal tissue of the affected side was incubated in the buffer on ice for 30 min and centrifuged at 12,000 g for 10 min. Then, BCA kit (Solarbio, Beijing, China) was used to measure the protein concentration of the supernatant. Afterwards, protein samples were split by 8% (GluN2B and m-calpain) or 15% (p38 MAPK and p-p38 MAPK) SDS-PAGE, and the electrophoretic voltage was 80 V to 120 V. The protein is then transferred to the PVDF membrane by a 200 mA current, and the action time was 70 min (p38 MAPK and p-p38 MAPK), 90 min (m-calpain), and 120 min (GluN2B), respectively. The following primary antibodies were used: Rabbit anti-GluN2B (65783, 1 : 1000, Abcam, USA); Rabbit anti-m-calpain (39165, 1 : 1000, Abcam, USA); Mouse anti-p38 (31828, 1 : 1000, Abcam, USA); Rabbit anti-p-p38 (4511, 1 : 1000, CST, USA); and Mouse anti-*β*-tubulin (HC101-01, 1 : 5000, TransGen, China). Secondary HRP-Conjugated Goat Anti-Rabbit or Goat Anti-Mouse antibody (1 : 5000, TransGen, China) was used. The optical density of the bands was determined by gel imaging system (Jena, Germany) with chemiluminescence reagent (Millipore, USA) as a developer solution. The intensity of chemiluminescence was measured using Visionworks 8.0.

### 2.9. Double Immunofluorescence Staining

Paraffin sections were dewaxed, rehydrated, antigen repaired (EDTA, Solarbio, C1034, China), and sealed with 5% goat serum (Solarbio, SL038, China) at room temperature for 30 min. The following primary antibodies were used: Rabbit anti-GluN2B (65783, 1 : 100, Abcam, USA); Rabbit anti-m-calpain (39165, 1 : 100, Abcam, USA); and Mouse anti-NeuN (104224, 1 : 100, Abcam, USA). The secondary antibody used was PE-labeled Goat Anti-Rabbit IgG (1 : 200, TransGen, China) and AF488-labeled Goat Anti-Mouse IgG (1 : 200, TransGen, China). The nuclei were labeled after incubated away from the light with DAPI for 2 min. Finally, images of sections were captured using a fluorescence microscope (Leica, Germany).

### 2.10. Statistical Analysis

SPSS21.0 was used for data processing and statistical analysis. The measurement data were expressed as the mean ± standard error. The measurement data at different time points were compared using one-way ANOVA for repeated measurement. The measurement data without time sequence was compared using one-way ANOVA or the Kruskal-Wallis test according to data distribution. *P* values of 0.05 were considered to indicate statistical significance.

## 3. Results

### 3.1. EA Pretreatment Induced Neuroprotection against Cerebral I/R

**Figure 2:**
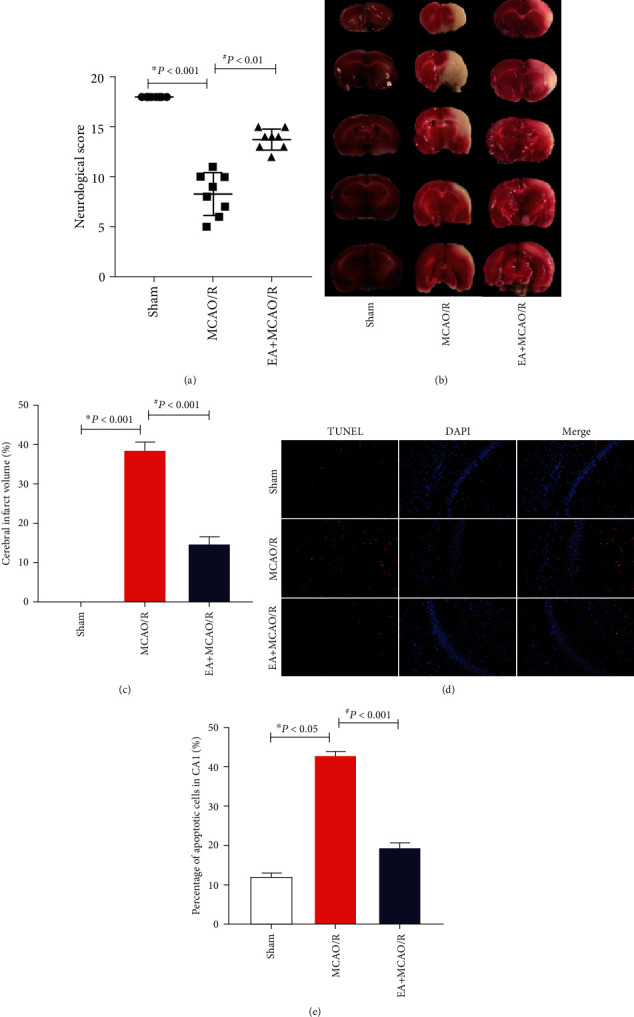
Effect of EA pretreatment on Garcia score, cerebral infarction volume, and the number of neuron apoptosis in the hippocampal CA1 region of MCAO/R rats. After cerebral I/R injury for 24 h, the neural behavior of the rats was evaluated by the Garcia score (*n* = 8), the infarct volume was detected by TTC staining of the brain slices (*n* = 4), and the cellular outcome was detected by TUNEL fluorescence staining (*n* = 4). (a) Comparison of Garcia score. The results conformed to the normal distribution yet with heterogenous variance. Differences among groups were examined using the Kruskal-Wallis test (*H* = 21.324, *P* < 0.001) followed by the Dunn test for post hoc multiple comparisons. (b) Representative slice of TTC staining. (c) Comparison of infarct volume. The results conformed to the normal distribution with homogeneous variance. Differences among groups were examined using AVONA (*F*(2, 9) = 513.909, *P* < 0.001) followed by LSD for post hoc multiple comparisons. (d) Representative immunofluorescence staining of TUNEL-positive cells (red) in brain sections. Scale bars = 100 *μ*m. (e) Comparison of apoptotic cell. The results conformed to the normal distribution and homogeneous variances. Differences among groups were examined using AVONA (*F*(2, 7) = 517.694, *P* < 0.001) followed by LSD for post hoc multiple comparisons. ^∗^*P* vs. sham; ^#^*P* vs. MCAO/R.

This EA pretreatment regime proposed based on TCM theory has never been explored for inducing cerebral ischemic tolerance. In order to evaluate the neuroprotective effect of this new EA pretreatment protocol on cerebral I/R, neurological behavior, infarct volume, and apoptosis in the hippocampal CA1 area were measured using Garcia score, TTC staining, and TUNEL fluorescence staining, respectively. The results confirmed the beneficial effect of this 5-day EA pretreatment on cerebral I/R injury. Compared with the sham group, rats of the MCAO/R model presented impaired neurological dysfunction (8.25 ± 2.12, *P* < 0.001; Figure 2(a)) and increased infarct volume (38.51 ± 2.21, *P* < 0.001; Figures 2(b) and 2(c)), and cells with positive TUNEL fluorescence suggested that apoptosis in the hippocampal CA1 area was also exacerbated in MCAO/R rats (42.76 ± 1.20, *P* = 0.019 < 0.05; Figures 2(d) and 2(e)). Meanwhile, pretreatment with EA might improve neurological deficit (13.75 ± 1.04, *P* = 0.001 < 0.01; Figure 2(a)), decrease infarct volume (14.57 ± 2.00, *P* < 0.001; Figures 2(b) and 2(c)), and reduce TUNEL-positive apoptotic cells in hippocampal CA1 area (19.33 ± 1.40, *P* < 0.001; Figures 2(d) and 2(e)).

### 3.2. EA Pretreatment Downregulated Neuronal Expression of GluN2B in Hippocampal CA1 Region Postcerebral I/R Injury

In order to test whether the glutamate proapoptotic receptor GluN2B might mediate the ischemic tolerance of EA pretreatment, overall expression of GluN2B in the hippocampus of the affected side was detected using WB, and the expression of GluN2B in neurons at the CA1 region was further observed using GluN2B and NeuN colabeling immunofluorescence staining.

**Figure 3:**
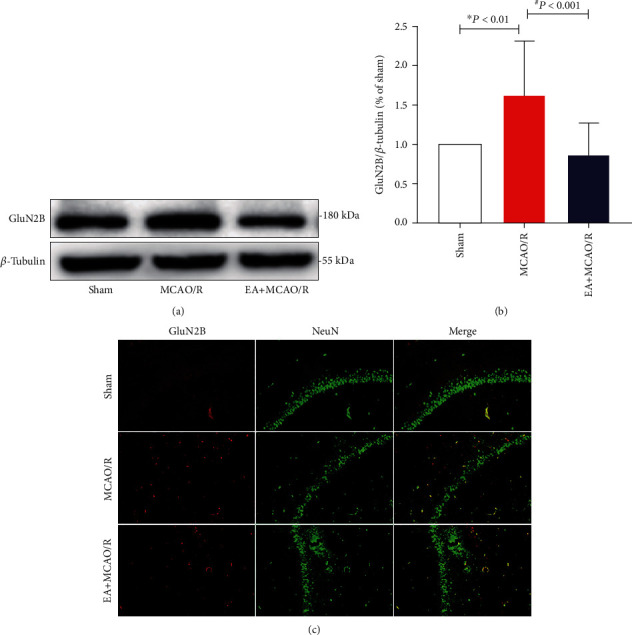
Effect of EA pretreatment on GluN2B expression (*n* = 4). GluN2B protein expression and cell localization were detected at 24 h after cerebral I/R by WB and double immunofluorescence staining. (a) Representative WB bands showing GluN2B expression in the rat hippocampus of three groups. (b) Comparison of the GluN2B expression. The results conformed to the normal distribution with heterogenous variance. Differences among groups were examined using the Kruskal-Wallis test (*H* = 18.598, *P* < 0.001) followed by the Dunn test for post hoc multiple comparisons. (c) Representative double immunofluorescence staining (yellow) of GluN2B-positive cells (red) and NeuN-positive cells (green) in brain sections. Scale bars = 100 *μ*m. ^∗^*P* vs. sham; ^#^*P* vs. MCAO/R.

The overall expression of GluN2B in the hippocampus of rats in the MCAO/R model group was significantly increased (1.62 ± 0.70, *P* = 0.002 < 0.01; Figures 3(a) and 3(b)) compared to sham rats, and EA pretreatment could reverse this reaction (0.86 ± 0.41, *P* < 0.001; Figures 3(a) and 3(b)). Moreover, when the labels of GluN2B and NeuN were merged, the number of colabeled cells in the EA+MCAO/R group was significantly lower than that in the MCAO/R group (Figure 3(c)). This suggested that EA pretreatment might inhibit the excessive activation of GluN2B receptor in neurons at the hippocampal CA1 region postcerebral I/R injury.

### 3.3. EA Pretreatment Downregulated the Neuronal Expression of m-Calpain in Hippocampal CA1 Region Postcerebral I/R Injury

In order to test whether m-calpain might also contribute to be the ischemic tolerance of EA pretreatment, overall expression of m-calpain in the hippocampus of the affected side was detected using WB, and the expression of m-calpain in the neurons at the CA1 region was further observed using m-calpain and NeuN double immunofluorescence staining.

**Figure 4:**
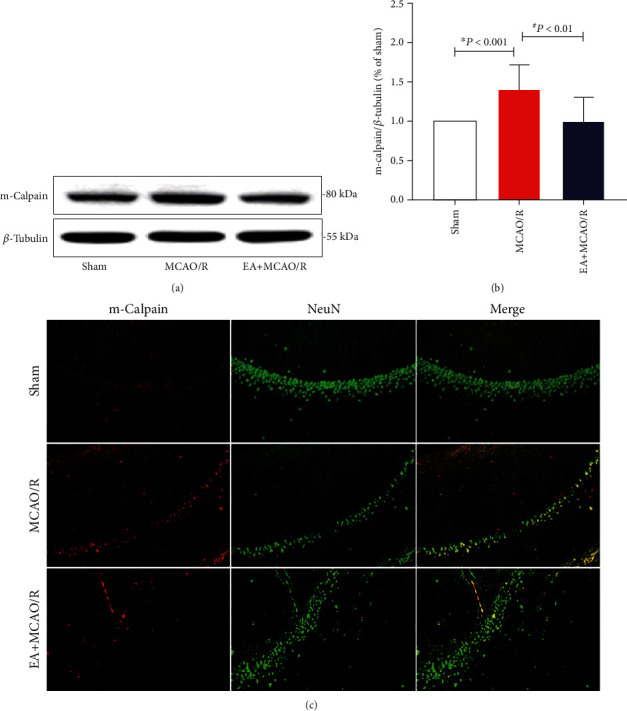
Effect of EA pretreatment on m-calpain expressed in the rat hippocampal CA1 neurons (*n* = 4). The expression and cell localization of m-calpain at 24 h after cerebral I/R were detected by WB and double-standard immunofluorescence staining. (a) Representative WB bands showing m-calpain expression in rats among the three groups. (b) Comparison of the m-calpain expression. The result did not conform to the normal distribution. Differences among groups were examined using the Kruskal-Wallis test (*H* = 21.421, *P* < 0.001) followed by the Dunn test for post hoc multiple comparisons. (c) Representative double immunofluorescence staining (yellow) of m-calpain-positive cells (red) and NeuN-positive cells (green) in brain sections. Scale bars = 100 *μ*m. ^∗^*P* vs. sham; ^#^*P* vs. MCAO/R.

It was found that overall expression of m-calpain in the hippocampus of rats in the MCAO/R group was significantly increased compared with the sham rats (1.40 ± 0.31, *P* < 0.001; Figures 4(a) and 4(b)), and overall expression of m-calpain was significantly decreased in the EA+MCAO/R group compared with that in the MCAO/R group (0.99 ± 0.31, *P* = 0.001 < 0.01; Figures 4(a) and 4(b)). m-Calpain and NeuN colabeled image showed the same intergroup change pattern (Figure 4(c)). This indicated that EA pretreatment might also inhibit the GluN2B downstream proapoptotic mediator m-calpain in the hippocampal neurons for rats' postcerebral I/R injury.

### 3.4. EA Pretreatment Downregulated the Phosphorylation of p38 MAPK in Hippocampal Region Postcerebral I/R Injury

Mitogen-activated protein kinases (MAPKs) are downstream targets of calpain the in neurons. After ischemic injury, activated m-calpain would activate p38 MAPK to initiate the cell apoptosis signaling pathway [17]. It was also a key issue that whether p38 MAPK activation might lead to ischemic tolerance of EA pretreatment. Therefore, the ratio of phosphate p38 to total p38 was also calculated after quantitation test using WB.

**Figure 5:**
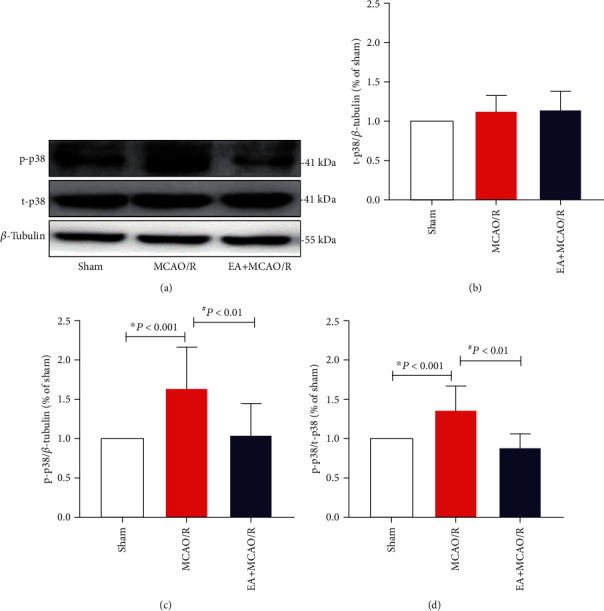
Effect of EA pretreatment on t-p38 and p-p38 expression in the hippocampal CA1 neurons (*n* = 4). The expression of p38 and p-p38 in the hippocampal neurons was detected by WB at 24 h after cerebral I/R. (a) Representative WB bands showing p38 MAPK expression in the rat hippocampus of three groups. (b, c) Comparison of total p38 (t-p38) and phophorylated p38 (p-p38) expression. The result of t-p38/*β*-tubulin conformed to normal distribution with homogeneous variance. Differences among groups were examined using AVONA (*F*(2, 33) = 1.941, *P* = 0.160 > 0.05). The result of p-p38/*β*-tubulin conformed to normal distribution with homogeneous variance. Differences among groups were examined using AVONA (*F*(2, 33) = 10.380, *P* < 0.001). (d) Comparison of p-p38/t-p38 ratio. The result of p-p38/t-p38 conformed to normal distribution with homogeneous variance. Differences among groups were examined using AVONA (*F*(2, 33) = 18.015, *P* < 0.001) followed by LSD for post hoc multiple comparisons. ^∗^*P* vs. sham; ^#^*P* vs. MCAO/R.

The expression of total p38 protein (t-p38) was stable among all groups (Figures 5(a) and 5(b)), but the level of phosphorylated p38 (p-p38) and the p-p38/t-p38 ratio were significantly different between each group. The expression and proportion of p-p38 in the hippocampal tissue of rats in the MCAO/R group was significantly higher than that in the sham group (1.59 ± 0.50, 1.49 ± 0.45, *P* < 0.001, *P* < 0.001; Figures 5(a), 5(c), and 5(d)), and the expression and proportion of p-p38 in the EA+MCAO/R group was significantly lower than that in the MCAO/R group (1.06 ± 0.37, 0.99 ± 0.34, *P* = 0.002 < 0.01, *P* = 0.001 < 0.01; Figures 5(a), 5(c), and 5(d)). This suggests that EA pretreatment may mitigate hippocampal cell apoptosis induced by excitatory neurotoxicity postcerebral I/R through inhibiting the phosphorylated activation of p38 MAPK.

## 4. Discussion

Proper preconditioning could protect against cerebral ischemia through activating several endogenous signaling pathways [8, 24]. It is applicable not only for people at high risk of stroke or stroke recurrence but also for patients with anticipated cerebral ischemia, such as invasive cerebral surgery. The anticipation of such preconditioning lies in reducing severity of ischemia injury, prolonging treatment window for other postattack intervention such as rt-PA, and promoting recovery capability [25]. Since the severity of neurological deficit is independently associated with an increased risk of symptomatic intracerebral hemorrhage post-t-PA, ischemic tolerance might also affect the occurrence of postthrombolysis hemorrhage through decrease severity of neurological deficit [26]. EA is considered as the combination of traditional Chinese medicine and modern electrical stimulation. Among all regimes of preconditioning, EA should be valued for its feasibility, practicability, and safety in clinical practice. Furthermore, acupuncture has already been widely used for poststroke management on motor, cognitive, and swallowing function [27–29]. It is highly possible that acupuncture could be recruited into the health delivery of systemic stroke management. Therefore, exploring the role and mechanism of EA in inducing cerebral ischemia is of great importance.

The majority of previous studies only stimulate GV20 for eliciting cerebral ischemic tolerance [11, 30, 31]. Our team proposed another acupuncture regime, i.e., stimulating GV20, SP6, and PC6 at the same time based on theory of traditional Chinese medicine (TCM). Ischemic tolerance could be induced with holistic adaptation through regulating spirit, activating collaterals, harmonizing *Qi* and blood, and nourishing the liver, spleen, and kidney. GV20, the most common used acupoint, mainly regulate spirit and activate local collaterals for ischemic tolerance. The addition of SP6 and PC6 could improve the efficacy in complementary of GV20 to fulfill the goal of holistic adaptation. Therefore, the combined EA protocol might present more stable and effective performance. It turned out that in comparison with the rats in MCAO/R, 5 days of repeated EA intervention on GV20, SP6, and PC6 could decrease neurological dysfunction, decrease infarct volume, and reduce apoptosis in hippocampal CA1 area. Therefore, this part of experiment could provide an effective EA prescription based on theory of TCM for further mechanism exploration and clinical efficacy validation. For the convenience of similar studies by other researchers who might be interested in cerebral ischemic tolerance, we listed all details of EA pretreatment in Methods, including acupoint location, electrical stimulation, and sign of *Qeqi*.

**Figure 6:**
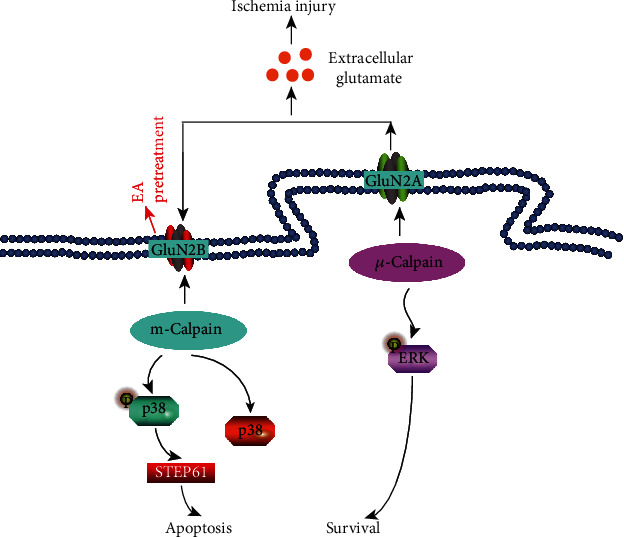
Bidirectional action of glutamate NMDA receptors during ischemic injury and role of EA pretreatment. GluN2B: the NR2B-containing NMDA receptors; GluN2A: the NR2A-containing NMDA receptors; STEP61: striatal-enriched protein tyrosine phosphatase; ERK: extracellular signal-regulated kinase.

When ischemic injury attacks, rapid release of glutamate from presynaptic neuron would induce excitotoxicity and finally leading to neuronal death [32], whereas neurotransmitters including glutamate have been validated as the mediators of acupuncture therapeutic effect in neurological diseases [33] and the activators of EA pretreatment in stimulating endogenous cerebral protection. Previous studies suggested that EA pretreatment can reduce glutamate concentration in the striatum [34] and hippocampus [35] of rats, respectively, in animal mode of cerebral I/R injury and vascular dementia. Reduction of extracellular glutamate by EA pretreatment might achieve through reabsorption by overexpression of glutamate transporter type 2 [36]. Neurotoxicity mainly depends on postsynaptic calcium overload. The NMDA subtype of glutamate receptors has attracted much attention in recent years due to its higher permeability to Ca^2+^ compared with voltage-gated channels when inducing calcium overload [1, 37]. Well-documented experimental evidence from both in vitro and in vivo models of stroke strongly supports that overactivation of the NMDA receptors is the primary step leading to neuronal injury after insults of stroke [38, 39]. As is shown in Figure 6, NR2A- and NR2B-containing NMDA receptor subtypes (GluN2B and GluN2A) have opposing roles in influencing the direction of synaptic plasticity when mediating cell death and cell survival in vivo rat model of ischemic stroke [39]. Namely, contrary to the role of neuroprotective GluN2A, activation of synaptic or extrasynaptic GluN2B receptor results in excitotoxicity, increasing neuronal apoptosis. Inhibition of NMDA receptor through intracranial [40] or intravenous [41] injection before ischemia can also reduce the infarct volume and prevent neuronal damage after cerebral ischemia. Furthermore, blocking GluN2B-mediated cell death was effective in reducing infarct volume only when the receptor antagonist was given before the onset of stroke [39]. Therefore, GluN2B could be a key to elicit cerebral ischemia tolerance. EA was found to improve cognitive impairment in rats through inhibiting GluN2B expression [42]. So, we tested the hypothesis that EA pretreatment might elicit cerebral tolerance to ischemia by regulating level of GluN2B using WB and double immunofluorescence staining. In concordance with the previous study [42], our results suggested that repeated EA before ischemia can inhibit GluN2B expression in hippocampal CA1 region. Therefore, glutamate proapoptotic receptor GluN2B might contribute to the reduction of post-I/R neural injury by mitigating excitotoxicity.

As is shown in Figure 6, apoptosis induced by GluN2B receptor is mediated by enzyme digestion reaction of m-calpain and the subsequent phosphorylation of the transcription factor [43]. Calpain activation is the intracellular calcium-dependent event with the greatest contribution to excitotoxicity [44]. When triggered by overloaded intracellular Ca^2+^, the Ca^2+^-dependent proteases m-calpain can cause degradation of cytoskeleton and structural proteins, and ultimately initiate the pathway leading to neuronal death [45]. Many previous studies had shown that focal cerebral ischemia can activate m-calpain in the hippocampus, cortex, and striatum through upregulating its protein expression at 1 h after cerebral ischemia [46], and then trigger calpain-mediated STEP (striatal-enriched protein tyrosine phosphatase) lysis and induce neurotoxicity [47]. Therefore, as the key downstream mediator of GluN2B, m-calpain activation is the most critical intracellular calcium-dependent event that is contributing to neurotoxicity [48]. At present, the regulatory effect of acupuncture on m-calpain has never been reported. Our study showed that m-calpain in the hippocampus was activated at 24 h after cerebral I/R injury, and EA pretreatment can decrease the expression of m-calpain in the hippocampal CA1 region neurons. This suggested that EA pretreatment might block the most important intracellular calcium activation pathway of excitotoxicity induced by I/R injury.

NMDA receptor-mediated death signaling is transcription-dependent [43]. The key transcription factor of GluN2B-mediated proapoptotic pathway is p38 MAPK [43]. A large number of studies had shown that the p38 MAPK signal transduction pathway is closely related to cerebral I/R injury and ischemia tolerance [49–51]. Level of phosphorylated p38 MAPK reached a peak at 24 hours after cerebral ischemia [52]. Previous studies had found that EA can reduce the relative density of phosphorylated p38 MAPK in an animal model of cerebral ischemia [51]. Our results showed that there was no marked difference in the expression level of the total p38 in each group at 24 h after cerebral I/R, but there was a significant difference in the ratio of phosphorylated p38 to total p38 in each group. The increased ratio in the model group indicated that p38 MAPK protein played a damaging role through phosphorylation activation after cerebral ischemia, which was consistent with previous studies. Our study also showed that EA pretreatment can inhibit p38 MAPK phosphorylation in the hippocampus in rats with cerebral I/R injury.

Some limitations also restrict interpretation of our study. Firstly, we only focused on the GluN2B-mediated proapoptotic pathway of excitotoxicity. However, the GluN2A-mediated prosurvival pathway also could contribute to the regulation of excitotoxicity. Previous study showed that EA pretreatment could also upregulate the expression of GluN2A [42]. Therefore, the synergic action of the GluN2B proapoptotic and GluN2A prosurvival pathways in excitotoxicity could be further elucidated in future studies. Secondly, the process of neurotoxicity postcerebral ischemia should be highly time-sensitive. So, further study should be conducted to illustrate the time-variant property of EA pretreatment on postischemic excitotoxicity from the perspective of extracellular glutamate.

## 5. Conclusion

This study suggested that our EA pretreatment regime could effectively increase the neurofunction and reduce the volume of cerebral infarction and the level of neuronal apoptosis in the hippocampal CA1 region for an animal model of cerebral I/R injury, and its mechanism may be related to the inhibition of the GluN2B/m-calpain/p38 MAPK proapoptotic pathway.

## Data Availability

The data used to support the findings of this study are available from the corresponding author upon request.
